# Molecular property prediction based on graph structure learning

**DOI:** 10.1093/bioinformatics/btae304

**Published:** 2024-05-06

**Authors:** Bangyi Zhao, Weixia Xu, Jihong Guan, Shuigeng Zhou

**Affiliations:** Shanghai Key Lab of Intelligent Information Processing, and School of Computer Science, Fudan University, Shanghai 200438, China; Shanghai Key Lab of Intelligent Information Processing, and School of Computer Science, Fudan University, Shanghai 200438, China; Department of Computer Science and Technology, Tongji University, Shanghai 201804, China; Shanghai Key Lab of Intelligent Information Processing, and School of Computer Science, Fudan University, Shanghai 200438, China

## Abstract

**Motivation:**

Molecular property prediction (MPP) is a fundamental but challenging task in the computer-aided drug discovery process. More and more recent works employ different graph-based models for MPP, which have achieved considerable progress in improving prediction performance. However, current models often ignore relationships between molecules, which could be also helpful for MPP.

**Results:**

For this sake, in this article we propose a graph structure learning (GSL) based MPP approach, called GSL-MPP. Specifically, we first apply graph neural network (GNN) over molecular graphs to extract molecular representations. Then, with molecular fingerprints, we construct a molecule similarity graph (MSG). Following that, we conduct GSL on the MSG, i.e. molecule-level GSL, to get the final molecular embeddings, which are the results of fuzing both GNN encoded molecular representations and the relationships among molecules. That is, combining both intra-molecule and inter-molecule information. Finally, we use these molecular embeddings to perform MPP. Extensive experiments on 10 various benchmark datasets show that our method could achieve state-of-the-art performance in most cases, especially on classification tasks. Further visualization studies also demonstrate the good molecular representations of our method.

**Availability and implementation:**

Source code is available at https://github.com/zby961104/GSL-MPP.

## 1 Introduction

The accurate prediction of molecular properties is a critical task in the field of drug discovery. By utilizing computational methods, this task can be accomplished with great efficiency, reducing both time and expense associated with identifying drug candidates. This is particularly important considering that the average cost of developing a new drug is currently estimated to be approximately $2.8 billion (Fleming 2018, Wieder *et al.* 2020) and the development period lasts a dozen of years, let alone the high risk of clinical failure (Sarkar *et al.* 2023). Naturally, a molecule can be abstracted as a topological graph, where atoms are treated as nodes and bonds are viewed as edges. In the past few years, deep graph learning methods, especially various graph neural networks (GNNs) have been applied in this field, offering effective molecular graph representations for accurate molecular property prediction (MPP) (Duvenaud *et al.* 2015, Song *et al.* 2020, Sun *et al.*, 2020). In GNNs, nodes iteratively update their representations after aggregating information from their neighbors and a final graph-pooling layer will generate a graph representation for the molecule. Up to now, various message passing layers have been proposed and applied, including GAT (Veličković *et al.* 2017), MPNN (Gilmer *et al.* 2017), and GIN (Xu *et al.* 2018). And later studies further considered to integrate edge features into the passing messages in order to improve the expressive power of their models, like DMPNN (Yang *et al.* 2019) and CMPNN (Song *et al.* 2020).

Despite the considerable progress, most of the recent studies focus only on the message passing within individual molecules. The relationships among molecules are often ignored, which could also play an important role in property prediction (Wang *et al.* 2021). A relatively easy and effective way is to construct a relationship graph among molecules using the structural similarity, because a critical assumption of medicinal chemistry is that structurally similar molecules tend to have similar biological activities (Johnson and Maggiora 1990). For example, fingerprint (carrying the structural information of the molecules) similarity search is often used in virtual screening (Muegge and Mukherjee 2016). However, this assumption is not always true since a phenomenon called activity cliff (AC) exits. An AC is defined as a pair of structurally similar compounds with a large potency difference against a given target (Maggiora 2006, Stumpfe and Bajorath 2012, Stumpfe *et al.* 2014, 2020). Thus, the relationship graph constructed by structural similarity may be not “perfect” for the downstream tasks. We need to take certain measures to enhance this relationship graph if we want to make full and proper use of it.

To address these problems above, we propose a novel two-level graph representation learning method for MPP, called GSL-MPP. Our method operates in a two-level molecular graph representation framework: (i) atom-level molecular graph representation where molecular graphs composed of atoms and bonds represent the intra-structures of molecules; and (ii) molecule-level graph representation where inter-molecule similarity graph (MSG in short) is constructed by fingerprint similarity to encode similarities between molecules that allows effective label propagation among similar molecules. Intra-molecular representation is done by GNNs, and inter-molecular representation is finished by graph structure learning (GSL). This two-level graph representation enables us to comprehensively exploit both intra-molecule and inter-molecule information to get better molecular representations and overcome (to some degree) the AC problem, consequently boosting MPP performance.

Specifically, we apply metric-based iterative GSL in our method. The MSG structure and molecular embeddings are updated for *T* times. During each iteration, GSL-MPP learns a better MSG structure based on better molecular embeddings, and in turn, learns better molecular embeddings with a better MSG structure. Besides, during the training process, we also add a GSL-specific loss to the common supervised loss for better MSG structure learning on both classification tasks and regression tasks. Our method is evaluated on 10 benchmark datasets including five classification tasks and five regression tasks. Experimental results show that our model can achieve state-of-the-art performance in most cases. Ablation studies show that the combination of fingerprint similarity and GSL is of particular effectiveness.

## 2 Materials and methods

### 2.1 Overview

**Figure 1.**
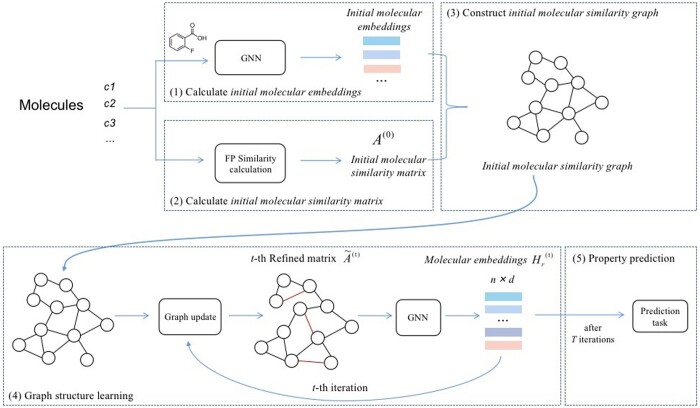
The workflow of GSL-MPP, which consists of five steps: (1) Calculating the *initial molecular embeddings* by encoding *molecule graphs* with a GNN. (2) Calculating the *initial molecular similarity matrix* with molecular feature vectors based on molecular fingerprints. (3) Constructing the *initial molecule similarity graph* (MSG) based on the above initial molecular embeddings and similarity matrix. (4) Performing structure learning on the MSG to iteratively updates the molecular embeddings and the graph structure, and eventually get the final molecular embeddings. (5) Doing property prediction with the final *molecular embeddings.*

The structure of our method GSL-MPP is illustrated in Fig. 1, which is operated on a two-level graph learning framework. Specifically, the two-level graph learning framework consists of (i) the *lower level*: atom-level molecular graphs encoded by GNN to extract the initial molecular representations, and (ii) the *upper level*: a molecule-level similarity graph, on which GSL is performed to iteratively learn the final molecular embeddings, where inter-molecular relationships are exploited, to get better and more accurate molecular embeddings for MPP.

The workflow of GSL-MPP is as follows: (i) Initial molecular embedding calculation: *molecule graphs* are first encoded by a GNN to obtain the *initial molecular embeddings*. (ii) Initial molecular similarity matrix calculation: molecules are represented as feature vectors using fingerprints, and then the *initial molecular similarity matrix* is calculated with the molecular feature vectors. (iii) Initial molecule similarity graph (MSG) construction: the *initial molecule similarity graph* is constructed, where each node is a molecule initially represented by the above GNN embeddings, and each edge attached with a weight—the similarity between the two corresponding molecules. (iv) Structure learning on MSG: GSL is performed on the MSG, which iteratively updates the molecular embeddings and the graph structure, to obtain the final molecular embeddings. (v) Property prediction: The final *molecular embeddings* are used for *property prediction*.

In what follows, we present the major techniques in GSL-MPP in detail, including molecular graph embedding, molecular GSL, loss function, and the algorithm.

### 2.2 Molecular graph embedding

Here, we describe how to represent a molecular graph as an initial vector by GNN. A molecule *m* can be abstracted as an attributed graph, where $G_{m}=(V,E)$, in which $\left|V\right|=n_{v}$ refers to a set of $n_{v}$ atoms (nodes) and $\left|E\right|=n_{e}$ refers to a set of $n_{e}$ bonds (edges) in the molecule. $x_{v}$ are used to represent the initial feature of node *v* and $N_{v}$ denotes the set of neighbors of node *v*.

#### 2.2.1 Node embedding

We use graph isomorphism network (GIN) (Xu *et al.* 2018) as intra-molecule GNN to extract each node’s embedding:

(1) $$h_{v}^{\left(k\right)}=MLP^{\left(k\right)}((1+\epsilon^{\left(k\right)})\cdot h_{v}^{\left(k-1\right)}+\sum_{u\in N(v)}h_{u}^{\left(k-1\right)}),$$

where MLP means multi-layer perceptron, $h_{v}^{\left(k\right)}$ is the representation vector of node *v* at the *k*th layer. We initialize $h_{v}^{\left(0\right)}=x_{v}$, and ϵ is a learnable parameter.

#### 2.2.2 Graph pooling

After gaining each node’s embedding, a READOUT operation is applied to get the initial molecular embedding $h_{g}$:

(2) $$h_{g}=\mathrm{READOUT}(\left\{h_{v}^{k}|v\in G\right\}|k=0,1,\ldots ,K).$$

### 2.3 Constructing MSG

Our inter-molecule graph reflects the relationships between molecules, where each node indicates a molecule, and each edge means the relationship between two molecules. As shown in Fig. 1, the initial feature vector of each node is the molecule’s embedding obtained by GNN (their embedding matrix is denoted as $X_{r}$), and the initial adjacency matrix $A^{\left(0\right)}$ is calculated by the structural similarity between molecules. Here, we calculate the structural similarity between molecules based on their Extended Connectivity Fingerprints (ECFP) (Morgan 1965).

ECFPs are circular fingerprints, possessing several beneficial characteristics: (i) they can be calculated fast; (ii) they are not predefined and can capture an almost limitless range of molecular characteristics including stereochemical information; (iii) they indicate the presence of specific substructures, facilitating interpretation of computation results (Rogers and Hahn 2010). Specifically, we get each molecule’s ECFP and calculate the Tanimoto coefficient as the similarity score. A hyperparameter $\epsilon_{tc}$ acts as a threshold to obtain a sparse matrix. That is, we mask off those elements in the adjacency matrix that are smaller than $\epsilon_{tc}$. We apply molecular fingerprints to construct $A^{\left(0\right)}$ because it contains useful structural information (Nguyen *et al.* 2023) and could offer an informative initial inter-molecule graph.

### 2.4 Structure learning on MSG

As we have discussed, this similarity graph constructed above may not be good enough for downstream tasks, therefore here GSL is employed to enhance the graph by exploiting inter-molecule relationships. Specifically, initial matrix built with fingerprint similarity only measure structural similarity between molecules and may not “perfectly” reflect true relationships among molecules, so we use GSL to refine the graph and consequently the molecular embeddings.

The core of GSL is the structure learner that could be grouped into three types: (i) metric-based approaches use a metric function like cosine similarity on pairwise node embeddings to calculate edge weights; (ii) neural approaches employ neural networks to infer edge weights; and (iii) direct approaches treat all elements of the adjacency matrix as learnable parameters (Zhu *et al.* 2021).

In this article, following IDGL (Chen *et al.* 2020), we adopt the metric-based approach and employ *m*-perspective weighted cosine similarity as the metric function:

(3) $$s_{ij}^{p}=\text{cos}(w_{p}⊙v_{i},w_{p}⊙v_{j}),\;s_{ij}=\frac{1}{m}\sum_{p=1}^{m}s_{ij}^{p},$$

where $s_{ij}^{p}$ estimates the cosine similarity between nodes $v_{i}$ and $v_{j}$, each perspective *p* considers one part of the semantics contained in the vectors and corresponds to a learnable weight vector $w_{p}$. The obtained $s_{ij}$ is the entry in row *i* and column *j* of the newly learned adjacency matrix *A*. Also the ϵ-neighborhood sparsification technique is applied to obtaining a sparse and non-negative adjacency matrix. Besides, this function requires $O(n^{2})$ complexity, so we need to address the scalability issue if the size of datasets becomes larger (see Section S3 of the Supplementary Material for more details).

The node embeddings $H_{r}$ and the adjacency matrix *A* will be alternately refined for *T* times. At the *t*th iteration, $A^{\left(t\right)}$ is calculated from the previously updated node embeddings $H_{r}^{\left(t-1\right)}$ by Eq. (3). Then we use the learned graph structure $A^{\left(t\right)}$ as supplementary to optimize the initial graph $A^{\left(0\right)}$:

(4) $${\tilde{A}}^{\left(t\right)}=\lambda A^{\left(0\right)}+(1-\lambda )\left\{\eta A^{\left(t\right)}+(1-\eta )A^{\left(1\right)}\right\},$$

where $A^{\left(1\right)}$ is the adjacency matrix learned from $X_{r}$ at the 1st iteration in order to maintain the initial node information. λ and η are hyperparameters.

After learning the adjacency matrix, we employ an *L*-layer inter-molecule GNN to learn node embeddings, and in the *l*th layer, $H_{r}^{\left(t,l\right)}$ is updated by

(5) $$H_{r}^{\left(t,l\right)}=\mathrm{ReLU}({\tilde{A}}^{\left(t\right)}H_{r}^{\left(t,l-1\right)}W_{r}^{\left(l\right)}),$$

$H_{r}^{\left(t\right)}=H_{r}^{\left(t,L\right)}$ is the final node embeddings in this iteration and $H_{r}^{\left(t,0\right)}=X_{r}$.

### 2.5 Loss function

After *T* rounds of iteration, the node (molecule) embeddings $H_{r}^{\left(T\right)}$ represent the final molecular representations. Based on this, predictions can be made for specific property $\hat{y}$ with a fully connected layer (FC) as follows:

(6) $$\hat{y}=FC(H_{r}^{\left(T\right)}).$$

The whole loss function used in our method consists of two parts: the label prediction loss and the GSL-specific loss. The label prediction loss function $L_{\mathrm{pred}}$ is obtained in a manner similar to existing methods:

(7) $$\;L_{\mathrm{pred}}=\ell (\hat{y},y),$$

where $\hat{y}$ represents the predicted value, *y* is the ground truth, and ℓ represents the loss function used. In classification tasks, it is the cross entropy loss, and in regression tasks, it is the mean squared error loss.

Since the quality of the learned inter-molecule graph structure is of great importance for our method, we further design a GSL-specific loss, hoping that the learned adjacency matrix does not contain wrong edges. We use $S_{\mathrm{train}}$ to represent molecules in training set and ${\tilde{A}}^{\left(T\right)}$ to represent the final adjacency matrix after being refined *T* times. In classification tasks, there exists a ground truth for the matrix, $A^{*}$($A_{ij}^{*}=1$ if $y_{i}=y_{j}$ else 0), i.e. molecules with the same label should be connected by edges. Thus, we define the GSL-specific loss as

(8) $$L_{\mathrm{GSL}}=\sum_{x_{i},x_{j}\in S_{\mathrm{train}}}{\left({\tilde{A}}_{ij}^{\left(T\right)}-A_{ij}^{*}\right)}^{2}.$$

However, in regression tasks, the prediction of a molecule is a real value and no native ground truth exists. We have to define it by ourselves. For the convenience of calculation, we only consider those molecular pairs with large difference (beyond a certain threshold $\epsilon_{y}$) in predicted values when calculating the GSL-specific loss:

(9) $$L_{\mathrm{GSL}}=\sum_{x_{i},x_{j}\in S_{\mathrm{train}}}{\left({\tilde{A}}_{ij}^{\left(T\right)}\right)}^{2},\;x_{i},x_{j}\;\mathrm{satisfy}\;\left|y_{i}-y_{j}\right|>\epsilon_{y}.$$

The whole loss function combines both the task prediction loss and the GSL-specific loss, that is $L=L_{\mathrm{pred}}+L_{\mathrm{GSL}}$.

### 2.6 Algorithm

The algorithm of our method is presented in Algorithm 1. After obtaining the initial molecular embeddings (Line 1) and constructing the initial inter-molecule similarity graph MSG (corresponding to the adjacency matrix) (Line 4), *T* iterations of GSL are applied to the MSG (Lines 5–13). During each iteration, the adjacency matrix is refined based on the node embeddings gained in the last iteration (Lines 6–7), while the node embeddings are updated based on the adjacency matrix obtained in the last iteration (Lines 9–11). MPP is done with the final molecular embeddings by a FC (Line 14).

Algorithm 1: The GSL-MPP algorithm

1: Obtain initial molecular embedding $h_{g,i}$ for each molecule $m_{i}$ by a graph-based molecular encoder (an intra-molecule GNN);

2: $X_{r}\leftarrow$ embedding matrix of all $h_{g,i}$;

3: $H_{r}^{\left(0\right)}\leftarrow X_{r}$;

4: Construct an MSG by the initial molecule similarity matrix $A^{\left(0\right)}$ using molecular fingerprint similarity;

5: **for**t=1 to *T* **do**

6:  Use GSL to learn a refined adjacency matrix $A^{\left(t\right)}$ by $H_{r}^{\left(t-1\right)}$ using Eq. (3);

7:  Combine initial and refined adjacency matrices $A^{\left(0\right)}$ and $A^{\left(t\right)},A^{\left(1\right)}$ to obtain ${\tilde{A}}^{\left(t\right)}$ by Eq. (4);

8:  Initialize node embeddings by $H_{r}^{\left(t,0\right)}=X_{r}$;

9:  **for**l=1 to *L* **do**

10:   Update node embedding $H_{r}^{\left(t,l\right)}$ by inter-molecule GNN using Eq. (5);

11:  **end for**

12:  $H_{r}^{\left(t\right)}\leftarrow H_{r}^{\left(t,L\right)}$;

13: **end for**

14: Obtain prediction $\hat{y}$ using $H_{r}^{\left(T\right)}$ by Eq. (6);

15: **if** in training phase **then**

16:  Calculate $L_{\mathrm{pred}}$ by Eq. (7) and $L_{\mathrm{GSL}}$ by Eq. (8) or Eq. (9) for $S_{\mathrm{train}}$;

17:  $L\leftarrow L_{\mathrm{pred}}+L_{\mathrm{GSL}}$;

18:  Back-propagate L to update model weights;

19: **end if**

## 3 Results 

Here, we present performance evaluation. First, we describe the experimental settings, including datasets used in experiments, baselines, performance metrics, and implementation details. Then, we introduce experimental results, including performance comparison with baselines, ablation studies, and visualization of embedding results. Due to space limit, we move some experimental results to the Supplementary Material.

### 3.1 Experimental setting

#### 3.1.1 Datasets

We use 10 benchmark datasets from MoleculeNet (Wu *et al.* 2018) in experiments, among which five are classification tasks and five are regression tasks. Specifically, BACE is about the binding results of several inhibitors; BBBP is the blood–brain barrier penetration dataset; SIDER, Clintox, Tox21 are three multi-task datasets corresponding to side effects or toxicity; ESOL, Lipophilicity, and Freesolv are regression datasets about physical chemistry properties. QM7 records electronic properties determined using *ab initio* density functional theory (DFT). QM8 contains computer-generated quantum mechanical properties.

Scaffold splitting of Yang *et al.* (2019) is adopted to split the datasets into training, validation, and test, with a 80%/10%/10% ratio, which is more empirical and challenging than random splitting. Following previous works (Rong *et al.*, 2020, Ma *et al.* 2022), we use three independent runs on three random-seeded scaffold splitting for each dataset.

#### 3.1.2 Baselines

We compare our method against 12 baselines. TF_Robust (Ramsundar *et al.* 2015) is a DNN-based multi-task framework that takes molecular fingerprints as input. GCN (GraphConv) (Duvenaud *et al.* 2015), Weave (Kearnes *et al.* 2016), and SchNet (Schütt *et al.* 2017) are three graph convolutional models. MPNN (Gilmer *et al.* 2017) and its variants MGCN (Lu *et al.* 2019), DMPNN (Yang *et al.* 2019), and CMPNN (Song *et al.* 2020) are models considering the edge features during message passing. AttentiveFP (Xiong *et al.* 2019) is an extension of the graph attention network. GROVER (Rong *et al.* 2020) and CoMPT (Chen *et al.* 2021) are two transformer-based models. Here, GROVER is compared without the pretrain process for a fair comparison. CoMPT is a transformer-based model utilizing both nodes and edges information in message passing process while CD-MVGNN (Ma *et al.* 2022) also constructs two views for atoms and bonds, respectively.

#### 3.1.3 Evaluation metrics

Following the evaluation criteria adopted by these baseline models, we use AUC-ROC to evaluate the performance of classification tasks. For regression tasks, RMSE is applied to evaluate FreeSolv, ESOL, and Lipophilicity while QM7 and QM8 are evaluated with MAE.

#### 3.1.4 Implementation details

Our model is implemented by Pytorch and Adam optimizer is used for model training. Other implementation details of our model are presented in Section S2 of the Supplementary Material.

### 3.2 Performance comparison

Tables 1 and 2 present the performance results of our model GSL-MPP and the baselines on the classification and regression datasets. Here, boldfaced values are the best results and italicized values are the 2nd best results. From the results, we have the following observations: (i) Compared to the current SOTA model CD-MVGNN, our model performs better on four of the five classification datasets with a 2.4% AUC lift on BBBP. Considering that our model is based on a simple GIN without complicated message passing procedures as used in CD-MVGNN and CoMPT, this result indicates the effectiveness of our GSL on the inter-molecule graph for prediction tasks. (ii) Our model achieves the best results on two of the five regression datasets and one 2nd best result on FreeSolv, which is not as good as CD-MVGNN, which may be because of the lack of real ground truth of relationship graphs in regression tasks. (iii) Our model gains the best result on ESOL and the 2nd best result on FreeSolv, which are small regression datasets with only 642 and 1128 labeled molecules, respectively. This confirms that GSL-MPP could help with the prediction for tasks with fewer labeled data since GSL-MPP is based on GNN and has relatively fewer parameters than transformer-style models like GROVER and CoMPT. (iv) In summary, our method achieves totally six best results and one 2nd best result on the 10 datasets, which shows that our model performs better in most cases than the existing models.

**Table 1.** Performance comparison between our model and baselines on classification datasets.

Model	BACE	BBBP	ClinTox	SIDER	Tox21
TFRobust	0.824 ± 0.022	0.860 ± 0.087	0.765 ± 0.085	0.607 ± 0.033	0.698 ± 0.012
GraphConv	0.854 ± 0.011	0.877 ± 0.036	0.845 ± 0.051	0.593 ± 0.035	0.772 ± 0.041
Weave	0.791 ± 0.008	0.837 ± 0.065	0.823 ± 0.023	0.543 ± 0.034	0.741 ± 0.044
SchNet	0.750 ± 0.033	0.847 ± 0.024	0.717 ± 0.042	0.545 ± 0.038	0.767 ± 0.025
MPNN	0.815 ± 0.044	0.913 ± 0.041	0.879 ± 0.054	0.595 ± 0.030	0.808 ± 0.024
DMPNN	0.852 ± 0.053	0.919 ± 0.030	0.897 ± 0.040	0.632 ± 0.023	*0.826 ± 0.023*
MGCN	0.734 ± 0.030	0.850 ± 0.064	0.634 ± 0.042	0.552 ± 0.018	0.707 ± 0.016
CMPNN	0.869 ± 0.023	0.929 ± 0.025	0.922 ± 0.017	0.617 ± 0.016	0.810 ± 0.022
AttentiveFP	0.863 ± 0.015	0.908 ± 0.050	0.933 ± 0.020	0.605 ± 0.060	0.807 ± 0.020
CD-MVGNN	*0.892 ± 0.011*	*0.933 ± 0.006*	*0.945 ± 0.037*	*0.639 ± 0.012*	**0.836 ± 0.006**
GROVER^a^	0.858	0.911	0.884	0.624	0.803
CoMPT	0.838 ± 0.035	0.926 ± 0.028	0.876 ± 0.031	0.612 ± 0.026	0.792 ± 0.020
Our model	**0.896 ± 0.007**	**0.957 ± 0.008**	**0.947 ± 0.020**	**0.652 ± 0.014**	0.800 ± 0.021

a GROVER does not use pretrained model for a fair comparison, and standard deviation is not provided in its original article.

**Table 2.** Performance comparison between our model and baselines on regression datasets.

Model	FreeSolv	ESOL	Lipop	QM7	QM8
TF_Robust	4.122 ± 0.085	1.722 ± 0.038	0.909 ± 0.060	120.6 ± 9.600	0.024 ± 0.001
GraphConv	2.900 ± 0.135	1.068 ± 0.050	0.712 ± 0.049	118.875 ± 20.219	0.021 ± 0.001
Weave	2.398 ± 0.250	1.158 ± 0.055	0.813 ± 0.042	94.688 ± 2.705	0.022 ± 0.001
SchNet	3.215 ± 0.755	1.045 ± 0.064	0.909 ± 0.098	74.204 ± 4.983	0.020 ± 0.002
MPNN	2.185 ± 0.952	1.167 ± 0.430	0.672 ± 0.051	112.960 ± 17.211	0.015 ± 0.002
DMPNN	2.177 ± 0.914	0.980 ± 0.258	0.653 ± 0.046	105.775 ± 13.202	*0.0143 ± 0.002*
MGCN	3.349 ± 0.097	1.266 ± 0.147	1.113 ± 0.041	77.623 ± 4.734	0.022 ± 0.002
CMPNN	2.060 ± 0.505	0.838 ± 0.140	*0.625 ± 0.027*	75.875 ± 12.360	0.015 ± 0.002
AttentiveFP	2.030 ± 0.420	0.853 ± 0.060	0.650 ± 0.030	126.690 ± 4.020	0.028 ± 0.001
CD-MVGNN	**1.552 ± 0.123**	*0.779 ± 0.026*	**0.553 ± 0.013**	*70.358 ± 5.962*	**0.0124 ± 0.001**
GROVER^a^	1.987	0.911	0.643	89.408	0.017
CoMPT	2.006 ± 0.628	0.822 ± 0.090	0.663 ± 0.035	72.165 ± 5.820	0.0148 ± 0.002
Our model	*1.974 ± 0.315*	**0.733 ± 0.071**	0.693 ± 0.063	**60.47 ± 4.094**	0.0196 ± 0.002

a GROVER does not use pretrained model for a fair comparison, and standard deviation is not provided in its original article.

### 3.3 Ablation study

To investigate the contribution of each component of our model, an ablation study is conducted on seven chosen datasets. We consider four variant models for comparison as follows:

- **Not any**: directly use $H_{r}^{\left(0\right)}$ to predict. It is almost a GIN network.
- **Only** $A^{\left(0\right)}$ : apply GNN on the initial molecule similarity graph $A^{\left(0\right)}$ constructed by ECFP similarity without GSL.
- **Only GSL**: use *de novo* GSL without an initial graph reference $A^{\left(0\right)}$.
- **No GSL-Loss**: use $A^{\left(0\right)}$ and GSL, but apply only the prediction loss.

Ablation results are given in Table 3. Here, we mainly consider the contribution of the initial adjacency matrix $A^{\left(0\right)}$ constructed by ECFP fingerprints and GSL process in our method. The results of “Not any,” “Only $A^{\left(0\right)}$,” and “No GSL-Loss” confirm that the use of $A^{\left(0\right)}$ could improve the performance of our model and the improvement will be much more significant when combined with GSL. Besides, it is interesting to notice that “Only GSL” often performs worse than “Not any,” which probably means learning an inter-molecule graph from scratch might be difficult and it is necessary for us to utilize the chemical information of molecular fingerprints to build an initial graph. Finally, while comparing “No GSL-Loss” and the complete “Our Model,” we can see that GSL-specific loss does make a difference for our method.

**Table 3.** Ablation study on four variants of our model.

	Classification (ROC-AUC)				Regression (RMSE)		
	BACE	BBBP	ClinTox	SIDER	FreeSolv	ESOL	Lipop
Not any	0.809 ± 0.032	0.943 ± 0.005	0.932 ± 0.012	0.593 ± 0.017	2.055 ± 0.405	0.962 ± 0.093	0.888 ± 0.046
Only A0	0.856 ± 0.049	0.951 ± 0.002	0.939 ± 0.033	0.639 ± 0.030	3.433 ± 0.552	0.945 ± 0.076	0.724 ± 0.040
Only GSL	0.850 ± 0.025	0.943 ± 0.017	0.875 ± 0.049	0.580 ± 0.021	2.638 ± 0.098	1.147 ± 0.256	0.899 ± 0.196
No GSL-loss	0.865 ± 0.034	0.953 ± 0.015	0.935 ± 0.016	0.651 ± 0.026	2.134 ± 0.155	0.821 ± 0.100	0.711 ± 0.047
Our model	**0.871 ± 0.038**	**0.957 ± 0.008**	**0.947 ± 0.020**	**0.652 ± 0.014**	**1.974 ± 0.315**	**0.799 ± 0.118**	**0.693 ± 0.063**

boldfaced values are the best results.

We also conduct experiments to show the results of using different values of some important hyperparameters on all the datasets. Table 4 reports the results of applying different values of λ, which is used to balance the learned graph structure and the initial graph structure. It can be seen that applying a large λ value (0.8 or 0.9) will generate a relatively good results on most datasets, which indicates the importance of the initial inter-molecule graph. Besides, Table 5 shows the impact of the number of iteration *T* on performance. We can see that as *T* increases from 1 to 5, performance on most datasets does not show continuous improvement, which means that the best *T* may be data dependent.

**Table 4.** Results for different λ values on different datasets.

Lambda	BACE	BBBP	ClinTox	SIDER	FreeSolv	ESOL	Lipop
0.1	0.702 ± 0.1	0.924 ± 0.006	0.914 + 0.026	0.564 + 0.035	2.812 + 0.239	0.983 + 0.094	0.714 + 0.056
0.3	0.846 ± 0.062	0.944 ± 0.008	**0.947 ± 0.020**	0.606 + 0.018	2.371 + 0.171	0.986 + 0.112	0.749 + 0.05
0.5	0.839 ± 0.043	0.921 ± 0.017	0.939 + 0.018	0.616 + 0.007	2.224 + 0.231	0.87 + 0.147	0.706 + 0.059
0.7	0.849 ± 0.049	0.942 ± 0.004	0.916 + 0.037	0.634 + 0.006	2.126 + 0.153	0.931 + 0.056	0.725 + 0.066
0.8	0.850 ± 0.028	**0.957 ± 0.008**	0.927 + 0.023	**0.652 ± 0.014**	**1.974 ± 0.315**	0.827 + 0.09	**0.693 ± 0.063**
0.9	**0.871 ± 0.038**	0.939 ± 0.013	0.925 + 0.023	0.643 + 0.004	2.279 + 0.089	**0.799 ± 0.118**	0.707 + 0.066

boldfaced values are the best results.

**Table 5.** Results for different *T* values on different datasets.

*T*	BACE	BBBP	ClinTox	SIDER	FreeSolv	ESOL	Lipop
1	0.848 ± 0.062	0.945 ± 0.007	0.93 ± 0.028	0.64 ± 0.017	2.724 ± 0.484	0.863 ± 0.046	0.718 ± 0.047
2	0.858 ± 0.045	0.923 ± 0.023	**0.947 ± 0.020**	0.636 ± 0.015	**1.974 ± 0.315**	0.86 ± 0.127	0.751 ± 0.053
3	0.857 ± 0.043	0.944 ± 0.008	0.93 ± 0.005	**0.652 ± 0.014**	2.182 ± 0.364	0.872 ± 0.087	**0.693 ± 0.063**
4	**0.871 ± 0.038**	**0.957 ± 0.008**	0.907 ± 0.018	0.64 ± 0.007	2.209 ± 0.41	0.861 ± 0.093	0.752 ± 0.047
5	0.851 ± 0.057	0.937 ± 0.011	0.918 ± 0.034	0.63 ± 0.015	2.309 ± 0.291	**0.799 ± 0.118**	0.751 ± 0.058

boldfaced values are the best results.

### 3.4 Visualization of molecular representations

**Figure 2.**
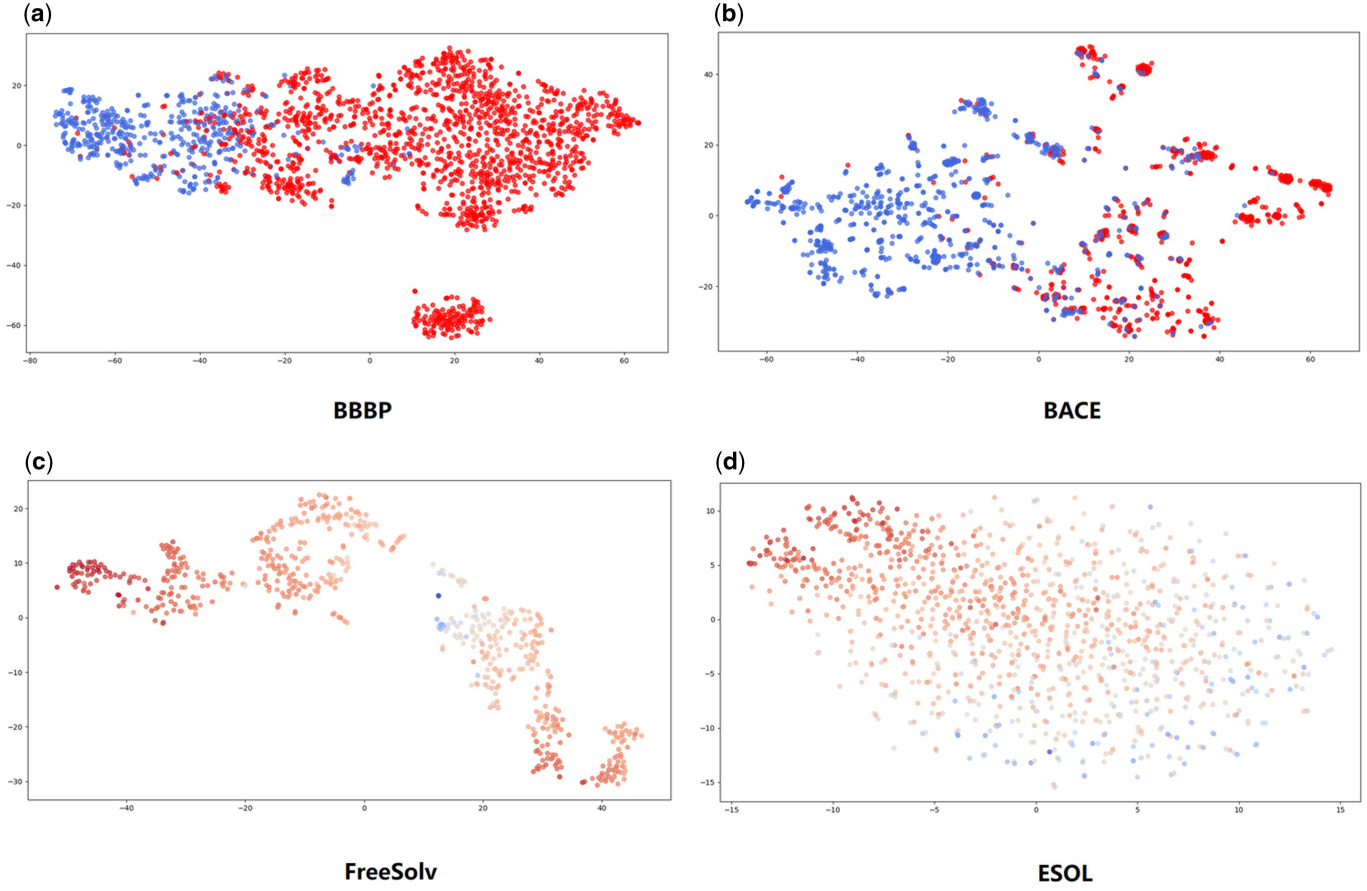
Visualization of molecular representations for four datasets: (a) BBBP, (b) BACE, (c) FreeSolv, and (d) ESOL. Each point represents a molecular representation in 2D space. For classification datasets BACE and BBBP, molecules of label 1 are colored in red and molecules of label 0 are colored in blue. For regression datasets FreeSolv and ESOL, the colors of the points change from red to blue as the property value increases.

To check the molecular representation learning ability of our model, we apply t-distributed Stochastic Neighbor Embedding (t-SNE) with default hyperparameters to visualizing the final molecular representations on four datasets, including two classification datasets (BACE and BBBP) and two regression datasets (FreeSolv and ESOL). t-SNE is a nonlinear dimensionality reduction technique for embedding high-dimensional data for visualization to a low-dimensional space (usually two or three dimensions). Specifically, it models each high-dimensional vector by a two- or three-dimensional point such that similar vectors are modeled as nearby points and dissimilar vectors are modeled as far-away points with high probability. Here, we embed the representations of molecules into a two-dimensional space through t-SNE (using the sklearn package in Python) and use the matplotlib package in Python to display it. The results are shown in Fig. 2.

We can see that molecules of different labels have a clear boundary for both two classification datasets, especially for BBBP. Molecules of the same label tend to be clustered together, while molecules of different labels are located apart. Also, there seems a certain distribution pattern existing among the molecules of different property values for the two regression datasets. For the FreeSolv dataset, molecules tend to move from the outer region to the inner region as the property value decreases. As for the ESOL dataset, molecules tend to move from upper left to lower right as the property value decreases. These results indicate that our model generates reasonable representations of molecules for downstream tasks.

## 4 Conclusion

In this article, we propose a new model based on two-level molecular representation for MPP. Unlike previous attempts focusing exclusively on message passing between atoms or bonds within individual molecule graphs, we further take use of the inter-molecule graph. Concretely, we utilize the chemical information of molecular fingerprints to construct an initial MSG, and employ GSL to refine the graph. Molecular embeddings based on GSL on the inter-MSG are used for MPP. Extensive experiments show that our model can achieve state-of-the-art performance in most cases, especially on the classification tasks. Ablation studies also validate the major designed components of the model.

However, there is still room to improve our model in the following directions: (i) Using more sophisticated graph-based models to encode molecular graphs rather than GIN. (ii) Designing new metrics other than weighted cosine similarity for GSL. (iii) Exploring new and more effective GSL methods.

## Supplementary Material

btae304_Supplementary_Data
