# Supplementary material for: Molecular property prediction based on graph structure learning
Source: Bioinformatics. 2024 May 6;40(5):btae304. doi: 10.1093/bioinformatics/btae304 (PMC11112045; doi:10.1093/bioinformatics/btae304)
Supplement: btae304_Supplementary_Data [file btae304_supplementary_data.pdf]

Molecular Property Prediction Based on Graph Structure Learning: Supplementary Materials

S1 Related Work

We review the related work from two aspects, molecular property prediction and graph structure learning.

S1.1 Molecular Property Prediction

Most methods for predicting molecular properties can be summarized using a general framework. In this framework, we first transform the input molecule m into a specific-length vector h using a representation function, $h = g(m)$. Then another prediction function is used to predict a specific property y based on h, $y = f(h)$. During this period, a good molecular representation is of vital importance to address molecular property prediction problems.

At early stages, traditional chemical fingerprints such as Extended Connectivity Fingerprints (ECFP) [9, 5] are used to encode a molecule to a vector. These fingerprints could carry the structural information of the molecules [10].

In order to improve the expressive power, recent works started to use the graph neural networks (GNNs) to acquire graph-level representation as molecular embedding. Examples include graph convolutional network (GCN) [3], graph attention network (GAT) [13], message passing neural network (MPNN) [4] and graph isomorphism network (GIN) [15].Later works extend the MPNN framework to consider bond information during message passing procedure, like DMPNN [16] and CMPNN [12]. Besides, CD-MVGNN [7] also considers both atom-level and bond-level message passing, and a cross-dependency mechanism is designed to ensure these two views rely on information from each other during feature updates, thereby enhancing expressive capabilities.

Recently, many efforts have also been made to integrate transformer to graph neural network. Molecule Attention Transformer(MAT) [8] attempts to incorporate node distance and graph structural information when calculating attention scores. Another work Grover [11] combines message-passing networks with the Transformer architecture to create a more expressive molecular encoder that captures information at two hierarchical levels. CoMPT [1] is also built upon the Transformer architecture. Unlike previous graph Transformer models that treated molecules as fully connected graphs, this approach employs a message

diffusion mechanism inspired by heat diffusion phenomena to integrate information from the adjacency matrix, alleviating the over-smoothing issue.

However, these methods only focus on the structure of a single molecule, while ignoring the important role of inter-molecular relationships for property prediction.

S1.2 Graph Structure Learning

The expressive power of GNNs often depends on the input graph structure. However, the initial graph structure is not always optimal for downstream tasks. On the one hand, the original graph is constructed from the original feature space, which may not reflect the "true" graph topology after feature extraction and transformation. On the other hand, errors can also occur when data is measured or collected, making the graph noisy or even incomplete. Graph structure learning (GSL) is one of the methods that can effectively solve this problem, through learning and optimizing the graph structure [17]. Recently, [2] proposed the method of iterative deep graph learning (IDGL) for jointly and iteratively learning graph structure and node embeddings in the field of natural language processing (NLP). It was later used by [14] for few-shot molecular property prediction. Compared to [14], our method is not based on few-shot situation and the datasets and baselines we choose are not for few-shot either. Besides, The specific implementation of GSL is different. More importantly, we try to construct an initial graph between molecules before we apply GSL, which is confirmed to be necessary in ablation study.

S2 Implementation Details

Our model apply a polynomial decay scheduler to the learning rate with two linear increase warm-up epochs and polynomial decay afterward. The power of polynomial decay is set to 1, indicating a linear decay. The final learning rate is 1e-9 and the max_epoch is 300. For the proposed model, on each dataset we try different hyper-parameter combinations, and take the hyper-parameter set with the best result. While building the initial inter-molecule graph, the radius of used ECFP is 2. The threshold of GSL-specific loss for regression tasks (ϵ_y) is 0.01. More details of the hyper-parameter setting in the implementation of our model are presented in Table S1.

Besides, anchor-based method described in S4 is applied during the evaluation of Tox21 and QM8 since they are multi-task datasets with many molecules.

S3 Case Studies

In order to further validate the effectiveness of GSL in our method, we conduct case studies on the BBBP and ESOL datasets. As we mentioned in the Introduction section of the main paper, structural similarity is not always equivalent to property similarity. For example, Fig. 1 (a) and (b) are two molecules from

Table S1: Hyper-parameter settings.

Hyper-parameter	Description	Value range
max_lr	maximum learning rate of polynomial decay scheduler	0.0001∼0.01
weight_decay	weight_decay weight decay percentage for Adam optimizer	0.00001∼0.001
gin_layers	number of the intra-molecule GIN layers	2∼5
gin_hidden_size	number of the hidden dimensionality in the intra-molecule GIN	32, 64, 128, 256
tc_epsilon	threshold of Tanimoto Coefficient for $A^{(0)}$ (ϵ_{tc})	0.0, 0.1, 0.2, 0.3, 0.5, 0.7
gsl_iter	number of the iterations for graph structure learning (T)	1∼5
gsl_gnn_layers	number of the inter-molecule GCN layers	2, 3
gsl_hidden_size	number of the hidden dimensionality in the inter-molecule GCN	32, 64, 128, 256
gsl_epsilon	threshold of similarity score in GSL (ϵ_{gsl})	0, 0,1, 0,2, 0.5
gsl_perspective	number of perspective used in GSL (m)	1, 2, 4, 8, 16
gsl_skip_conn	the ratio of initial matrix while updating the graph structure (λ)	0.1, 0.3, 0.5, 0.7, 0.9
gsl_update_ratio	the ratio of t-th learned matrix while updating the graph structure (η)	0.1, 0.3, 0.6, 0.8. 1.0
dropout	dropout rate	0, 0.1, 0.2, 0.4, 0.6
gsl_coff	the coefficient of the GSL related loss (μ)	0.1, 0.3, 0.5, 0.7, 0.9

the BBBP dataset. They differ greatly in structure: the molecule of Fig. 1(a) has one ester group, one benzene structure and two chlorine substituents while the molecule in Fig. 1(b) is a fatty chain substituted barbituric acid. The fingerprint (ECFP) similarity between them is very low (0.068). However, both of these two molecules could penetrate the blood-brain barrier (BBB) and thus have the same classification label because they both have a relatively long side chain. After using GSL, the similarity score between these two molecules in the newly learned matrix is 0.709, showing similar abilities to penetrate BBB and significantly helping MPP. Such examples can also be found in the ESOL dataset, which records the water solubility data of molecules. Fig. 1(c) and (d) are two fatty chains, which only differ in the number of carbon atom. The ECFP similarity between them is 1.00 because they have same substructure. However, the number of carbon atoms has a huge effect on the solubility in this situation. We can see that the logS (log solubility measured in mol/L) of the chain in Fig. 1(c) is -7.96, which is more than one hundred times of the chain in Fig. 1(d). To deal with this problem, we use GSL to learn a new similarity score 0.115, which is more reasonable. These examples demonstrate that by using GSL, we can get better and more accurate molecule embeddings, so consequently boosting the performance of MPP.

S4 Scaling Our Model to Larger Datasets

During GSL, the similarity metric function calculate similarity scores for all pairs of graph nodes, which requires $\mathcal{O}(n^2)$ complexity. So we need to address the scalability issue if the size of datasets becomes larger. Following IDGL [2], we apply an anchor-based method. During each iteration, We learn a node-anchor similarity matrix $R \in \mathbb{R}^{n\times s}$ instead of the original complete adjacency matrix $A \in \mathbb{R}^{n\times n}$. s represents the number of anchor nodes, which is a hyperparameter that can be set according to different datasets. By using R instead of A, the time and space complexity can be reduced from $\mathcal{O}(n^2)$ to $\mathcal{O}(ns)$. Therefore, Eq.

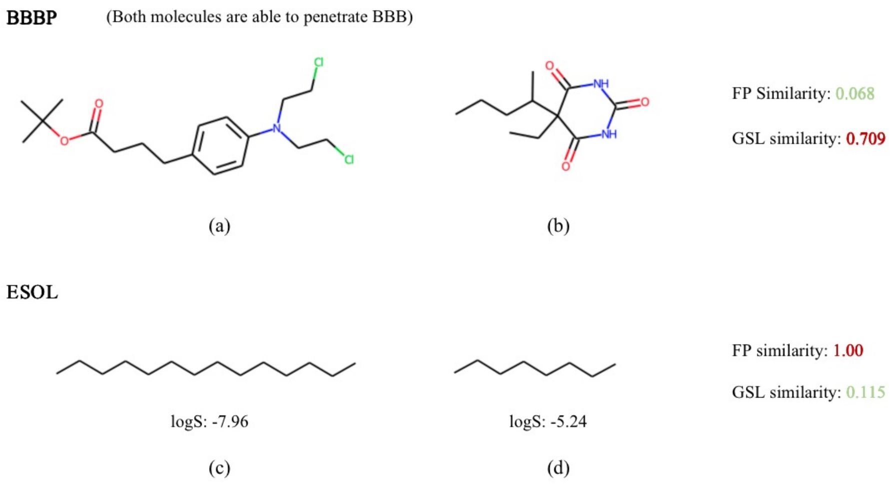

Figure 1: Case studies on BBBP and ESOL

(3) in the paper can be rewritten as the following:

$$s_{ik}^p = \cos(w^p \odot v_i, w^p \odot u_k), \quad s_{ik} = \frac{1}{m}\sum_{p=1}^{m} s_{ik}^p \tag{1}$$

where s_{ik} is the similarity score between node v_i and anchor u_k. The procedure of message passing should also be changed accordingly. The node-anchor similarity matrix R allows only direct connections between nodes and anchors. We call a direct travel between a node and an anchor as one-step transition described by R. Based on theories of stationary Markov random walks, we can actually recover A from R by calculating the two-step transition probabilities.

Using the above anchor-based GSL, We firstly evaluate whether introducing anchor nodes will have a great impact on the original prediction performance of our model. Results are given in Table S2. We can find that anchor-based GSL performs a little worse than the original GSL in these molecule datasets but the performance degradation is not significant. So we think it is appropriate for us to apply anchor-based GSL in larger-scale molecule datasets.

After completing the above evaluation, we test the anchor-based GSL method on the HIV dataset which includes over 40000 molecules and compare it with some existing models. Except for CD-MVGNN, the results of other models on the HIV data set are from PharmHGT [6]. PharmHGT is a recently proposed model based on the Transformer structure, which treats molecules as heterogeneous graphs. The ROC-AUC of CD-MVGNN on the HIV data set is obtained experimentally by ourselves. Results are given in Table S3. Our method is able to achieve the optimal ROC-AUC on the HIV dataset, showing that after introducing anchor nodes, our method can be well extended to larger-scale datasets and achieve satisfactory results.

Table S2: Performance comparison between original and anchor-based GSL.

	BACE	BBBP	ClinTox	SIDER	FreeSolv	ESOL	Lipop
Origin	0.865	0.953	0.935	0.651	2.134	0.821	0.711
Anchor-based	0.818	0.949	0.95	0.612	2.208	0.794	0.747

Table S3: Performance comparison between our model (using anchor-based GSL) and baselines.

	ROC-AUC%
Our model	**81.8**
PharmHGT	80.6
DMPNN	78.6
CD-MVGNN	78.4
CoMPT	78.1
CMPNN	77.4
AttentiveFP	75.7
MPNN	74.1
GROVER	62.5

References

[1] Jianwen Chen, Shuangjia Zheng, Ying Song, Jiahua Rao, and Yuedong Yang. Learning attributed graph representations with communicative message passing transformer. *arXiv preprint arXiv:2107.08773*, 2021.

[2] Yu Chen, Lingfei Wu, and Mohammed Zaki. Iterative deep graph learning for graph neural networks: Better and robust node embeddings. *Advances in neural information processing systems*, 33:19314–19326, 2020.

[3] David K Duvenaud, Dougal Maclaurin, Jorge Iparraguirre, Rafael Bombarell, Timothy Hirzel, Alán Aspuru-Guzik, and Ryan P Adams. Convolutional networks on graphs for learning molecular fingerprints. *Advances in neural information processing systems*, 28, 2015.

[4] Justin Gilmer, Samuel S Schoenholz, Patrick F Riley, Oriol Vinyals, and George E Dahl. Neural message passing for quantum chemistry. In *International conference on machine learning*, pages 1263–1272. PMLR, 2017.

[5] Robert C Glen, Andreas Bender, Catrin H Arnby, Lars Carlsson, Scott Boyer, and James Smith. Circular fingerprints: flexible molecular descriptors with applications from physical chemistry to adme. *IDrugs*, 9(3):199, 2006.

[6] Yinghui Jiang, Shuting Jin, Xurui Jin, Xianglu Xiao, Wenfan Wu, Xiangrong Liu, Qiang Zhang, Xiangxiang Zeng, Guang Yang, and Zhangming Niu. Pharmacophoric-constrained heterogeneous graph transformer model

for molecular property prediction. *Communications Chemistry*, 6(1):60, 2023.

[7] Hehuan Ma, Yatao Bian, Yu Rong, Wenbing Huang, Tingyang Xu, Weiyang Xie, Geyan Ye, and Junzhou Huang. Cross-dependent graph neural networks for molecular property prediction. *Bioinformatics*, 38(7):2003–2009, 2022.

[8] Łukasz Maziarka, Tomasz Danel, Sławomir Mucha, Krzysztof Rataj, Jacek Tabor, and Stanisław Jastrzębski. Molecule attention transformer. *arXiv preprint arXiv:2002.08264*, 2020.

[9] H. L. Morgan. The generation of a unique machine description for chemical structures-a technique developed at chemical abstracts service. *Journal of Chemical Documentation*, 5(2):107–113, 1965.

[10] Ngoc-Quang Nguyen, Gwanghoon Jang, Hajung Kim, and Jaewoo Kang. Perceiver cpi: a nested cross-attention network for compound–protein interaction prediction. *Bioinformatics*, 39(1):btac731, 2023.

[11] Yu Rong, Yatao Bian, Tingyang Xu, Weiyang Xie, Ying Wei, Wenbing Huang, and Junzhou Huang. Self-supervised graph transformer on large-scale molecular data. *Advances in Neural Information Processing Systems*, 33:12559–12571, 2020.

[12] Ying Song, Shuangjia Zheng, Zhangming Niu, Zhang-Hua Fu, Yutong Lu, and Yuedong Yang. Communicative representation learning on attributed molecular graphs. In *IJCAI*, volume 2020, pages 2831–2838, 2020.

[13] Petar Veličković, Guillem Cucurull, Arantxa Casanova, Adriana Romero, Pietro Lio, and Yoshua Bengio. Graph attention networks. *arXiv preprint arXiv:1710.10903*, 2017.

[14] Yaqing Wang, Abulikemu Abuduweili, Quanming Yao, and Dejing Dou. Property-aware relation networks for few-shot molecular property prediction. *Advances in Neural Information Processing Systems*, 34:17441–17454, 2021.

[15] Keyulu Xu, Weihua Hu, Jure Leskovec, and Stefanie Jegelka. How powerful are graph neural networks? *arXiv preprint arXiv:1810.00826*, 2018.

[16] Kevin Yang, Kyle Swanson, Wengong Jin, Connor Coley, Philipp Eiden, Hua Gao, Angel Guzman-Perez, Timothy Hopper, Brian Kelley, Miriam Mathea, et al. Analyzing learned molecular representations for property prediction. *Journal of chemical information and modeling*, 59(8):3370–3388, 2019.

[17] Yanqiao Zhu, Weizhi Xu, Jinghao Zhang, Yuanqi Du, Jieyu Zhang, Qiang Liu, Carl Yang, and Shu Wu. A survey on graph structure learning: Progress and opportunities. *arXiv e-prints*, pages arXiv–2103, 2021.
